# Repercussions of adjuvant-induced arthritis on body composition, soleus muscle, and heart muscle of rats

**DOI:** 10.1590/1414-431X20198969

**Published:** 2020-03-02

**Authors:** L.M. Pita, M.A. Spadella, M.C. Montenote, P.B. Oliveira, A.B. Chies

**Affiliations:** 1 Laboratório de Farmacologia, Faculdade de Medicina de Marília, Marília, SP, Brasil; 2 Laboratório de Embriologia Humana, Faculdade de Medicina de Marília, Marília, SP, Brasil; 3 Departamento de Farmacologia, Instituto de Biociências de Botucatu, Botucatu, SP, Brasil

**Keywords:** Experimental arthritis, Cachexia, Musculoskeletal, Microcirculation, Cardiovascular, Body composition

## Abstract

This study investigated the repercussions of adjuvant-induced arthritis (AIA) on body composition and the structural organization of the soleus and cardiac muscles, including their vascularization, at different times of disease manifestation. Male rats were submitted to AIA induction by intradermal administration of 100 μL of *Mycobacterium tuberculosis* (50 mg/mL), in the right hind paw. Animals submitted to AIA were studied 4 (AIA4), 15 (AIA15), and 40 (AIA40) days after AIA induction as well as a control group of animals not submitted to AIA. Unlike the control animals, AIA animals did not gain body mass throughout the evolution of the disease. AIA reduced food consumption, but only on the 40th day after induction. In the soleus muscle, AIA reduced the wet mass in a time-dependent manner but increased the capillary density by the 15th day and the fiber density by both 15 and 40 days after induction. The diameter of the soleus fiber decreased from the 4th day after AIA induction as well as the capillary/fiber ratio, which was most evident on the 40th day. Moreover, AIA induced slight histopathological changes in the cardiac muscle that were more evident on the 15th day after induction. In conclusion, AIA-induced changes in body composition as well as in the soleus muscle fibers and vasculature have early onset but are more evident by the 15th day after induction. Moreover, the heart may be a target organ of AIA, although less sensitive than skeletal muscles.

## Introduction

Rheumatoid arthritis (RA) is an autoimmune disease affecting about 1% of the world population (1). This chronic and progressive disease is characterized by an inflammatory response that first affects the articular structures (2,3). The RA-related inflammatory process that begins in the joints triggers systemic manifestations (4), which are characterized by extra-articular injuries in the early or later stages of the disease (5,6).

The systemic manifestations of RA can occur in different organs and tissues (4,7). Changes in the total body mass, as well as in the masses of both skeletal and adipose tissue, are described in RA patients. These changes may lead to rheumatoid cachexia, characterized by loss of muscle mass, with or without fat mass change (8,9). Evidence suggests that body mass loss is directly related to severity and RA mortality (8). Although rheumatoid cachexia is a well-studied clinical manifestation in RA patients due to its impact on the health of these patients, many questions remain. It is not yet fully established whether the reduction of muscle mass in these patients is consequent to a decrease in the number of muscle fibers or to the loss of proteins that constitute these fibers. Details are also not known about the vascular changes that can occur in the muscles affected by arthritis.

Pathophysiological mechanisms similar to those observed in rheumatoid cachexia have also been described in experimental models of arthritis (10–12). Like in RA, experimental arthritis reduces both muscle mass and the total body mass of animals (10–13). We decided to use the adjuvant-induced arthritis (AIA) model, which presents rapid clinical evolution, is very reproducible, and shows similarities to RA (14,15). Because it is an experimental model, AIA also enables an invasive approach to the manifestations of arthritis in these animals.

In addition, arthritis-induced cardiovascular manifestations have also been described in recent years (6,16–19). The mortality rate is 50% higher in RA patients than in the general population (20 [Bibr B21]). Research has demonstrated in humans and animal models that arthritis can affect both macro- (16,18,20–22) and microcirculation (6,19,23,24). RA-induced injuries in the heart muscle may occur regardless of hemodynamic changes and/or cardiovascular risk factors that might be associated (25,26). Moreover, evidence suggests that repercussions of arthritis on the microcirculation precede those occurring on vessels of conductance (6,19). However, it is still necessary to understand in greater depth the changes induced by arthritis on the vascularization of skeletal and cardiac musculature. Finally, little information is available about the temporal evolution of the changes induced by arthritis on the skeletal and cardiac muscles.

Hence, the present study aimed to investigate the AIA repercussions on body composition and on the structural organization of the soleus and cardiac muscles, including their vascularization, at different times of arthritis manifestation.

## Material and Methods

### Animals

Seventy male Wistar rats (12 weeks old) were used. During the experiments, the animals were housed in a room next to the laboratory, inside cages (50×40×20 cm) with four animals per cage, under controlled temperature (21-24°C), 12-h light/dark cycle, with food and water *ad libitum*. This study was approved by the Research Ethics Committee on the Use of Animals of Marilia Medical School/CEUA-FAMEMA (protocol number 158/17).

### Experimental groups

The animals were distributed into the following experimental groups: Control (CTRL): false-immunized; AIA4: immunized and studied 4 days after AIA induction; AIA15: immunized and studied 15 days after AIA induction; AIA40: immunized and studied 40 days after AIA induction. To minimize the influence of seasonal differences, the control animals were subdivided into three subgroups, each studied in parallel to the AIA groups (AIA4, AIA15, and AIA40) to analyze body mass gain. For the remaining analyzes, however, these three subgroups were regrouped into a single CTRL group.

### Adjuvant-induced arthritis (AIA) protocol

Under anesthesia with 2,2,2–tribromoethanol (250 mg/kg, *ip*), rats were submitted to intradermal injection of 100 μL emulsion of mineral oil-distilled water (3:1) containing 50 mg/mL heat-inactivated *Mycobacterium tuberculosis* (Difco, USA), in the right hind paw. The CTRL animals received only the emulsion (false-immunized). After AIA induction, the animals were returned to their cages with food and water *ad libitum*. These animals were observed daily after AIA induction. The AIA-induced articular inflammatory process was first detected in the hind paws of these animals by erythema and edema. The edema was quantified by measuring the diameter of the tibiotarsal joint (hind paw diameter) using an analog pachymeter (0.05 mm accuracy). Animals submitted to AIA that showed negative C-reactive protein (CRP) were excluded from the study.

### Food intake

Throughout the experimental protocol period, food intake was estimated daily. Therefore, each cage containing four animals received 500 g of chow daily. Twenty-four hours later, the amount of chow remaining in each cage was weighed. The food intake was calculated by the following equation (27):

$$Food intake=\frac{offered food (g) – remaining food (g)}{number of animals in the cage}$$

### Sample harvest

The animals were weighed, euthanized by deep thiopental anesthesia (Thiopentax^®^, 10 mg/100 g of body weight, *ip*, Cristália - Produtos Químicos Farmacêuticos Ltda., Brazil) and then exsanguinated through puncture of the inferior vena cava. The blood harvested was placed in a tube containing coagulation activator and then centrifuged (1613 *g*, 10 min, 4°C) to obtain the serum. The serum volume recovered was aliquoted and stored at -80°C for further analysis.

Soleus muscle of both hind paws and heart were also harvested from these animals and weighed. The wet mass of soleus muscles and heart were normalized by the tibia length (cm) and body mass (kg) of each animal, respectively. Later, these tissues were fixed for 24 h in 4% paraformaldehyde solution (prepared in PBS), with pH adjusted to 7.2. Then, these tissues were washed in running water for 24 h. The tissues remained immersed in 70% alcohol until processing.

### Body composition

Body composition was estimated based on the lean mass and fat mass of these animals. Lean mass was estimated by summing the wet mass of soleus, gastrocnemius, and extensor digitorum longus muscles, harvested in both legs, normalized by the tibia length (cm). Fat mass was estimated by summation of the periepididymal and retroperitoneal adipose tissue, normalized by the tibia length (cm) (28).

### C-reactive protein

C-reactive protein (CRP) was determined in the serum of the animals by the RCP-LÁTEX kit (Ebram Produtos Laboratoriais Ltda, Brazil), according to the manufacturer's instructions.

### Histopathological analysis

For histological analysis, soleus and cardiac muscles fixed in 4% paraformaldehyde were dehydrated in 95% ethanol and embedded in a Leica Historesin Embedding Kit^®^ (Leica Biosystems, Germany). The 5-µm-thick sections were stained with hematoxylin and eosin. Digital photomicrographs were obtained using the Olympus CellSens image capture software (Olympus Corp., Japan).

### Morphometric-stereological analyses

For each soleus and cardiac muscle, 10 histological fields at 1000× magnification were randomly captured to measure the diameter (µm) of muscle cells, using Olympus CellSens software. From the same histological field, the number of muscle cells and capillaries was counted in a fixed total area of 14226.51 µm^2^. Capillary and fiber densities consisted of the average number of capillaries and fibers, respectively, per histological fields captured in each studied soleus muscle.

The number of arterioles was also determined at 400× magnification from digital images of intentional histological fields of the soleus and cardiac muscles per rat in a fixed total area of 88741.73 µm^2^, using the same Olympus software.

The ratio of capillary density to muscle cell density was determined for the soleus and heart muscles, according to the equation:

$$Capillary/fiber ratio=\frac{Capillary density}{Fiber density}$$

### Statistical analysis

The parametric distribution of the data was verified by the Shapiro-Wilk test. If parametric distribution was found, the comparisons between the groups were made by one-way analysis of variance (one-way ANOVA), followed by Tukey's post-test. In these cases, data are reported as means±SE. Differences were considered statistically significant if P≤0.05.

When parametric distribution was found to be violated, comparisons between the groups were made using the non-parametric Kruskal-Wallis test. In these cases, the Mann-Whitney test was used, with P values adjusted by Holm-Sidak (P≤0.017), for peer-to-peer comparison. Non-parametric data are reported as median and interquartile ranges (25-75%).

All data analyses were performed using SPSS^®^ software (IBM, USA), version 19.0.

## Results

### Paw diameter

**Figure 1.**
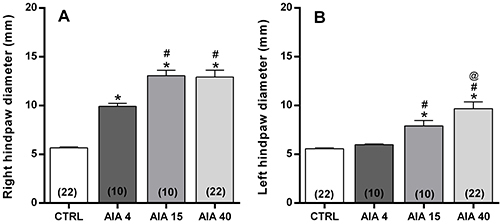
Right (**A**) and left (**B**) hind paw diameter determined in control (CTRL) animals or in those submitted to adjuvant-induced arthritis (AIA), 4 days (AIA4), 15 days (AIA15), and 40 days (AIA40) after induction. Data are reported as means±SE. The number of independent samples is reported in parentheses. *P<0.0001 *vs* CTRL; ^#^P<0.05 *vs* AIA4; ^@^P≤0.05 *vs* AIA15 (one-way ANOVA, followed Tukey's *post*-test).

The AIA induced a time-dependent increase in the animals' hind paw diameter. In the right hind paw, this diameter increase was statistically significant in relation to the control group from the 4th day, reaching fullness at 15 days after AIA induction (Figure 1A). On the other hand, in the left hind paw, the increase in diameter reached statistical significance on the 15th day and continued to increase until the 40th day after AIA induction (Figure 1B).

### Body composition

**Figure 2.**
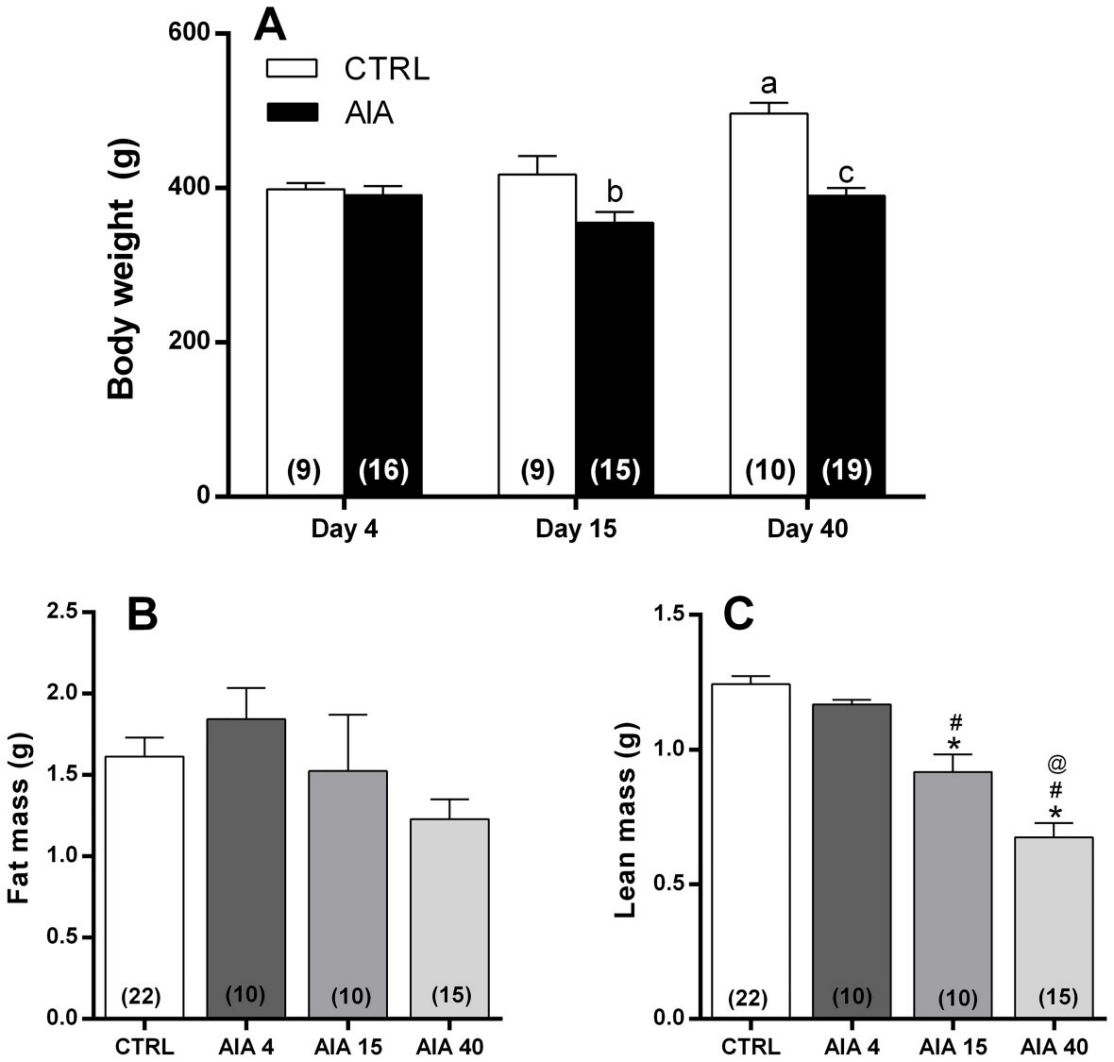
Values of body weight (**A**), fat mass (**B**), and lean mass (**C**) determined in control (CTRL) animals or in those submitted to adjuvant-induced arthritis (AIA) 4 days (AIA4), 15 days (AIA15), and 40 days (AIA40) after induction. Data are reported as means±SE. The number of independent samples is reported in parentheses. ^a^P<0.01 *vs* CTRL assessed on the 4th day after false-immunized; ^b^P≤0.05 *vs* CTRL assessed on the 15th day after false-immunized; ^c^P<0.01 *vs* CTRL assessed on the 40th day after false-immunized. *P<0.01 *vs* CTRL; ^#^P≤0.05 *vs* AIA4; ^@^P<0.01 *vs* AIA15 (one-way ANOVA, followed Tukey's *post*-test).

Unlike the CTRL animals, the AIA animals showed no gain of body mass throughout the evolution of the disease (Figure 2A). In this manner, the mean body mass of AIA animals was significantly lower in relation to their controls, both 15 and 40 days after AIA induction. There was no significant difference in fat mass between the groups (Figure 2B). On the other hand, AIA induced a time-dependent lean mass reduction in the studied animals. Consequently, mass values were significantly lower in AIA15 compared to the CTRL and AIA4 groups, as well as in the AIA40, compared to all other studied groups (Figure 2C).

### Food intake

AIA reduced food consumption, but only on the 40th day after induction. Food consumption in the AIA40 group (37.90; 26.70-43.30 g) was significantly lower (P<0.017; Kruskal-Wallis test followed by Mann-Whitney for peer-to-peer comparison) than both CTRL (57.65; 51.25-62.35 g) and AIA15 groups (59.0; 43.75-63.33 g), but not the AIA4 group (52.50; 42.50-62.50 g).

### Histopathological analysis

**Figure 3.**
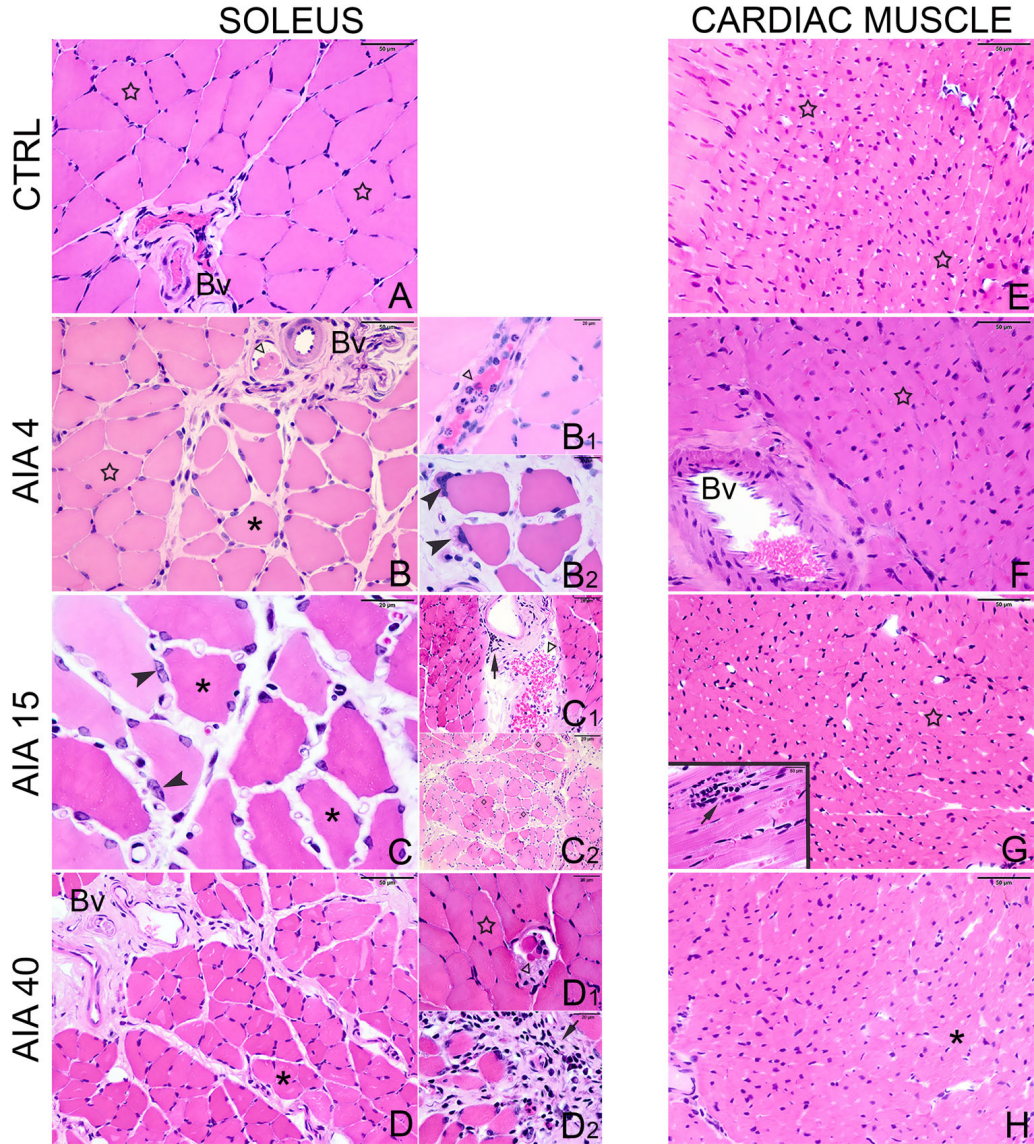
Photomicrographs of soleus (**A**-**D**) and cardiac (**E**-**H**) muscles taken from control (CTRL) animals or those submitted to adjuvant-induced arthritis (AIA) 4 days (AIA4), 15 days (AIA15), and 40 days (AIA40) after induction. In the control groups, the soleus and cardiac muscles exhibited normal morphology (**A**, **E**). In the AIA groups, at all times after induction, the soleus muscle showed signs of damage (**B**-**B**_2,_ **C**-**C**_2,_ **D**-**D**_2_). In the cardiac muscle, AIA promoted inflammatory infiltrate 15 days after induction (**G**, **G**-inset) and apparent atrophy in cardiomyocytes 40 days (**H**) after induction compared with AIA4 (**F**). BV: blood vessels; star: normal muscle fibers; non-filled arrowhead: leukocyte migration; asterisk: atrophied fibers; arrowhead: nuclear aggregation; arrow: inflammatory infiltrate. Staining: hematoxylin and eosin. Magnification bars: **A**, **B**, **D**-**H**, 50 μm; **C**, **B**_1_-**D**_1,_ **B**_2_-**D**_2_, 20 μm; **G-inset**, 50 μm.

In the histopathological analysis, fibers of the soleus muscles taken from CRTL animals presented normal and classic histological organization (Figure 3A). On the other hand, AIA-induced alterations occurred at all studied times. In the AIA4 group, bundles of muscle fibers were composed of both atrophic and normal fibers. The massive presence of leukocytes inside blood vessels with a predominance of neutrophils was also observed. Some muscle fibers had nuclear aggregation in the peripheral region (Figure 3B-B2). On the 15th day after induction, the muscle bundles had fibers with greater atrophy and edema in the interstitial area. The presence of leukocytes remained, characterizing intense inflammatory infiltration. Some muscle fibers also exhibited an irregular shape and dilation. Fibers with nuclear aggregation were also observed (Figure 3C-C2). Atrophied fibers, inflammatory edema in the perimysium, and the presence of neutrophils also occurred in the soleus muscles taken from AIA40 animals, but with smaller intensity and frequency. However, these animals showed a greater presence of mononuclear cells in the inflammatory infiltrate. In addition, several muscle bundles exhibited a normal pattern (Figure 3D-D2).

The AIA-induced morphological alterations were later observed in cardiomyocytes. The cardiomyocytes taken from AIA rats 4 days after induction and CTRL animals had a normal and classic pattern (Figure 3E and F). On the 15th day after induction, in spite of inflammatory infiltrate areas among the cardiomyocytes, these cells exhibited a normal structure (Figure 3G, G-inset). On the 40th day after induction, the majority of the cardiac muscle regions presented a normal organization, but in some areas, the cardiomyocytes exhibited an apparent atrophic shape (Figure 3H).

### Biometric and morphometric-stereological analyses

AIA reduced the wet mass of the soleus muscle in a time-dependent manner. This reduction in wet mass was significant on the 4th day, but was more evident on the 40th day after AIA induction. Moreover, the number of arterioles in the soleus muscle was significantly increased 15 days after AIA induction. This increase was no longer observed 40 days after AIA induction. Thus, AIA15 animals had more arterioles compared to CTRL and AIA40 animals. In parallel, increased capillary density was observed in the soleus muscle of AIA15, compared to CTRL animals. In addition, soleus muscles taken from AIA15 and AIA40 animals had an increased fiber density, in parallel with a significant reduction of diameter. Actually, the reduction in the diameter preceded the increase in fiber density, being observed from the 4th day after AIA induction. Moreover, the capillary/fiber ratio in the soleus muscle was reduced on the 15th day, reaching statistical significance compared to the control group on the 40th day after AIA induction (Table 1).

**Table 1.** Morphometric-stereological analyses of the soleus and heart muscles after AIA (adjuvant-induced arthritis) induction at 4, 15, and 40 days.

Parameters	CTRL	AIA4	AIA15	AIA40
Soleus muscle				
Wet mass (g)	0.10±0.002 (21)	0.08±0.006* (10)	0.07±0.005* (10)	0.05±0.004**^#+^ (15)
Arteriole number (n)	12.67±3.3 (9)	24.27±4.4 (11)	32.14±6.8*^+^ (7)	12.00±2.9 (7)
Capillary density	115.5 (109.3-125.5) (8)	137.5 (119.3-158.3) (10)	213** (142-241) (7)	148 (122-169) (7)
Fiber density	57±5.42 (7)	68±4.56 (7)	127±16.7*^#^ (7)	137±15.7*^#^ (7)
Fiber diameter (µm)	45.75±1.57 (7)	37.65±1.50* (7)	26.14±1.69*^#^ (7)	25.36±1.49*^#^ (6)
Capillary/fiber ratio	2.22±0.14 (9)	2.26±0.14 (10)	1.68±0.17^#^ (7)	1.11±0.09*^#^ (7)
Heart				
Wet mass (g)	3.63±0.09 (21)	3.51±0.11 (10)	3.61±0.17 (10)	3.94±0.13 (15)
Arteriole number (n)	20.11±2.9 (9)	18.11±2.7 (9)	16.17±4.2 (6)	27.63±5.9 (8)
Capillary density	159 (130.5-321.5) (9)	164 (128.5-183) (9)	258.5 (224.5-337.3) (6)	169 (117.5-213.8) (8)
Cardiomyocyte density	345±36.1 (5)	280±30.6 (5)	285±35.3 (5)	410±52.7 (5)
Cardiomyocyte diameter (µm)	15.71±0.62 (5)	17.53±1.78 (5)	13.61±0.62 (5)	12.68±1.26^#^ (5)
Capillary/cardiomyocyte ratio	0.84±0.20 (5)	0.69±0.10 (5)	0.92±0.02 (5)	0.46±0.07 (5)

Data are reported as means±SE. The number of independent samples is reported in parentheses. Comparisons by one-way ANOVA, followed by Tukey's *post*-test. Data of capillary density are reported as median and interquartile ranges (25-75%). Comparisons by non-parametric Kruskall-Wallis test, followed by peer comparisons by the Mann-Whitney test. *P<0.05 *vs* CTRL; **P<0.017 *vs* CTRL; ^#^P<0.05 *vs* AIA4; ^+^P<0.05 *vs* AIA40.

In parallel, AIA did not promote significant changes in the wet mass of the heart in any of the studied groups. Modifications in both arteriole number and capillary density were not observed in the cardiac muscle. In addition, although the cardiomyocyte density was slightly higher in the AIA40 group, compared to the other groups, this difference was not significant. The diameter of the cardiomyocytes in the AIA40 animals, however, was less than the AIA4 animals but not the CTRL group. Finally, no difference between groups was found in the capillary/fiber ratio in cardiac muscle (Table 1).

## Discussion

The presented data reinforce the concept that arthritis is a systemic disease, with repercussions that go far beyond the joints (4,29). In addition to the already well-characterized joint inflammatory process, AIA animals exhibited a loss of wet mass, atrophy of cardiomyocytes as well as atrophy of muscle fibers and microvascular changes in soleus muscle.

To understand the temporal evolution of AIA, we observed the animals at three specific times. The first observation was made 4 days after AIA induction, a period considered pre-clinical by some authors (6,18). At this time, the inflammatory joint process existed, although it was still monoarticular, without inflammatory signs in the hind paw contralateral to the immunization. Not even the inflammatory process of the ipsilateral hind paw was complete at this time, since the volume of these paws reached its peak on the 15th day, remaining equally high until the 40th day after AIA induction.

Animals were also examined 15 days after AIA induction, when arthritis becomes polyarticular. The articular inflammatory process is active at this time, with evident edema and increased blood flow in the joints of these animals (6). Also around the 15th day, AIA becomes a systemic disease. Febrile peaks are reported in the AIA animals between the 13th and 17th day after induction (30), when the animals begin to develop severe bone deformities and edema worsens in their joints. To characterize this later stage of the model, the animals were also studied 40 days after AIA induction.

Unlike the CTRL, all AIA animals presented positive CRP (data not shown), which indicates the presence of an inflammatory process (25,31–33). This also indicates that the AIA-induced inflammatory process was already present on the 4th day, although this is considered a pre-clinical phase (6). Moreover, although the AIA-induced inflammatory process is still monoarticular on the 4th day, its systemic repercussions are already present.

The AIA animals had less body mass gain than the CTRL animals over the 40 days of the experimental protocol. Less body weight gain as a consequence of AIA has also been reported in previous studies performed in rats (10,11,27). Reductions in body mass may be associated with decreased locomotion, reduced food intake, metabolic changes, and increased skeletal muscle proteolysis (10,11,27).

A lower food intake should be considered when analyzing weight losses in experimental models or clinical situations characterized by discomfort and/or motor limitations. A reduction in food intake was detected only at 40 days after AIA induction, when the animals presented generalized edema and deformation in their paws. This may have led to a loss of mobility (11), with consequent reduction in the search for food. A reduction in food intake has also been observed in rats 21 days after AIA induction (10,27,34). This indicates that the reduction in food intake observed on the 40th day after AIA induction started earlier, sometime after the 15th day. In the present study, however, no reduction in food intake occurred on the 15th day after AIA induction, although there was already reduced body mass. Thus, at least at 15 days after AIA induction, the animals were not in an anorexia or malnutrition condition. Indeed, there was also no reduction in fat mass 15 days after AIA induction.

Our results reinforce the hypothesis of a greater proteolysis in the AIA animals, at least on the 15th day after induction. These animals had a significant reduction in lean mass, along with reduced wet mass and diameter of the soleus muscle fibers. This process seems to worsen in the later phase of the model when the animals also reduced food intake. A previous study found AIA-induced reduction in rat gastrocnemius muscle mass, concomitantly with a decrease in the cross-sectional area of both fast and slow fibers (10). According to these authors, this muscle mass reduction may involve an increase in the production of pro-inflammatory cytokines and is more intense around the 22nd day after AIA induction, although some ubiquitin ligases, such as muscle RING-finger protein-1 (MuRF-1) and atrogin-1, are more expressed around the 16th day. Reduction of soleus and rectus femoris muscle mass was also observed in rats submitted to AIA (*Mycobacterium tuberculosis*)-induced monoarticular arthritis. This muscle mass reduction, which involved a decrease in the cross-sectional area of both fast and slow fibers, was already seen around the 7th day after AIA induction (35). Loss of mass in both the gastrocnemius and tibialis anterior muscles and reduced mobility have also been reported in mice submitted to collagen-induced arthritis (CIA), but from the 45th day after the first immunization (10–12,35).

In soleus muscles, induced AIA reduced the diameter of fibers, as well as the increment of their density. These data suggest that the AIA-induced reduction of skeletal muscle mass was mainly due to atrophy of muscle fibers and not so much to a reduction in their number. Notably, the reduction of both soleus wet mass and the diameter of its fibers was significant on the 4th day after AIA induction. The animals' lean mass reduction, however, was significant only from the 15th day after AIA induction. This suggests that AIA effects on the soleus muscle preceded its effects on the gastrocnemius and/or extensor digitorum longus muscles, both taken into account in the calculation of lean mass.

The histopathological analysis of the soleus muscle reinforced that atrophy of at least part of the muscle fibers already occurred 4 days after AIA induction. This atrophy was accompanied by massive infiltration of polymorphonuclear cells, thereby suggesting the participation of an acute inflammatory process. Some muscle fibers also presented nuclear aggregation in peripheral regions. This modification may indicate cytoskeletal proteolysis (36) perhaps related to the muscle atrophy that was ongoing. On the 15th day after AIA induction, the atrophy becomes even more evident, corroborating the histomorphometric and stereological findings. At this stage, both intense inflammatory infiltrate and edema persisted. In addition, some fibers had irregular shape and/or nuclear aggregation. In the later phase of the model, at 40 days after AIA induction, the inflammatory infiltrate was less intense. Moreover, mononuclear cells could also be observed, which corroborated the resolution of the inflammatory process in this model (6,37).

AIA also increased the number of arterioles and the capillary density in the soleus muscles. These changes occurred 15 days after AIA induction, but disappeared in the later phase of the model. These vascular changes may reflect a body response to mitigate the AIA-induced muscle mass loss. In fact, increased microcirculation may be a mechanism to attenuate muscle atrophy by disuse, since it improves tissue perfusion (38). In this regard, the observed muscle mass loss could have been potentiated by the reduction of locomotion, since decreased mobility has already been described in animals affected by AIA (39,40).

Nevertheless, AIA induced a reduction in the capillary/fiber ratio in the soleus muscle of these animals, beginning 15 days after induction. This indicated that AIA did not increase the number of capillaries in the muscle, but only increased the number of capillary-fiber ensembles per field as a consequence of the reduced diameter of the fibers. This may also explain the observed increase in the number of arterioles. Thus, in the muscle as a whole, there was no increase in vascularization. Moreover, the increase in the number of arterioles and capillary density, observed on the 15th day, was reverted on the 40th day after AIA induction. Interestingly, the capillary/fiber ratio decreased even more in soleus muscles collected on the 40th day after AIA induction. This suggests that, instead of augmentation, there was a reduction of vascularization in the later phase of the model. More specifically, atrophy of the fibers occurred first, and then the vascularization of the fibers was reduced.

In hearts, no significant AIA-induced changes of wet mass occurred at any of the times studied. However, the heart wet mass assessment was not always able to detect subtle structural modifications of the cardiac musculature. On the other hand, the diameter of cardiomyocytes in the AIA40 animals was slightly smaller than in the AIA4 animals. Notably, no significant difference was observed between AIA40 and CTRL animals. Possibly, because this difference was within the limits of statistical significance, it was only detected in relation to the AIA4 group that presented mean values slightly higher than the CTRL group. This reduction did not imply an increase of the density of cardiomyocytes within the muscle. The number of arterioles, capillary density, and capillary/fiber ratio in this musculature also did not significantly change. Nevertheless, AIA-induced injuries in the heart muscles were confirmed by slight histopathological changes, characterized mainly by the presence of inflammatory infiltrate among cardiomyocytes that were evident mainly 15 days after the induction of AIA. These data suggest that the cardiac musculature, although less sensitive than the skeletal muscles, is not completely free from AIA effects.

Finally, the data presented here reinforced the hypothesis that the manifestations of arthritis may have different temporal evolutions in various organs and systems. Therefore, the presented data have great therapeutic interest, since they may support future studies that seek ways to approach in advance the manifestations of RA that develop over the course of the disease.

The present study showed that the AIA-related systemic inflammatory process began within the first few days after its induction, even before arthritis becomes polyarticular. The AIA-induced changes in the body composition were more evident from the 15th day after induction and tended to aggravate over time. Histopathological modifications could be observed in the soleus muscles by the 4th day and were more evident 15 days after AIA induction. By the 15th day, AIA-induced structural changes characterized by muscle fibers atrophy and vascular densification became evident. In addition, cardiac muscle also exhibited slight AIA-induced histopathological changes. This suggested that the heart may be a target organ of AIA, although less sensitive than skeletal muscles.
